# The Bone Niche of Chondrosarcoma: A Sanctuary for Drug Resistance, Tumour Growth and also a Source of New Therapeutic Targets

**DOI:** 10.1155/2011/932451

**Published:** 2011-05-22

**Authors:** E. David, F. Blanchard, M. F. Heymann, G. De Pinieux, F. Gouin, F. Rédini, D. Heymann

**Affiliations:** ^1^INSERM, UMR 957, Physiopathologie de la Résorption Osseuse et Thérapie des Tumeurs Osseuses Primitives, Faculté de Médecine, 1 rue Gaston Veil, 44035 Nantes Cedex 1, 44035 Nantes, France; ^2^Université de Nantes, Nantes Atlantique Universités, Laboratoire de Physiopathologie de la Résorption Osseuse et Thérapie des Tumeurs Osseuses Primitives, 44035 Nantes, France; ^3^University Hospital, Hôtel Dieu, CHU de Nantes, 44035 Nantes, France; ^4^EA3855, University Hospital, 2 bd Tonnelle, 37044 Tours Cedex, France; ^5^University Hospital, Hôpital Trousseau, CHRU de Tours, 37042 Tours Cedex, France

## Abstract

Chondrosarcomas are malignant cartilage-forming tumours representing around 20% of malignant primary tumours of bone and affect mainly adults in the third to sixth decade of life. Unfortunately, the molecular pathways controlling the genesis and the growth of chondrosarcoma cells are still not fully defined. It is well admitted that the invasion of bone by tumour cells affects the balance between early bone resorption and formation and induces an “inflammatory-like” environment which establishes a dialogue between tumour cells and their environment. The bone tumour microenvironment is then described as a sanctuary that contributes to the drug resistance patterns and may control at least in part the tumour growth. The concept of “niche” defined as a specialized microenvironment that can promote the emergence of tumour stem cells and provide all the required factors for their development recently emerges in the literature. The present paper aims to summarize the main evidence sustaining the existence of a specific bone niche in the pathogenesis of chondrosarcomas.

## 1. Introduction

Most chondrosarcomas (90%) are conventional chondrosarcomas which occur in the medullar cavity or at the bone surface. The fact that cartilaginous tumours are mainly observed in bones formed from endochondral ossification strengthens the relationship between the differentiation of normal chondrocytes and these neoplastic cells. Chondrosarcoma cells are cytologically and phenotypically related to the different chondrocyte subtypes observed in the growth plate, and all cell shapes can be observed in the tumour mass [1–4]. Thus, these similarities are in favour of a mesenchymal stem-cell origin for chondrosarcoma cells [1, 5]. The development of cancer cells in bone site responds to several biological mechanisms potentially applicable to numerous other entities. For instance, invasion of bone by a primary or metastatic tumour cell affects the balance between early bone resorption and bone formation. This dysregulation of osteoblast-osteoclast coupling induces the release of factors initially trapped in the bone matrix, which in turn promote tumour cell proliferation [6]. Thus, the bone tumour microenvironment controls the tumour growth and is also described as a sanctuary that contributes to drug resistance patterns [7]. The specific and different bone sites in which the various sarcomas are able to grow reinforce the prominence of the tumour microenvironment. Chondrosarcomas are also characterized by their chemo- and radioresistance leading to a therapeutic surgical approach which remains the only available treatment with a 10-year survival between 30% and 80% depending on the grade [8, 9]. Currently, surgical excision is the main treatment for all chondrosarcoma subtypes [10], and nonsurgical treatments of their microenvironment are under investigation. In this context, a better understanding of the bone niche which interacts with chondrosarcoma is one of the future therapeutic options. The present paper aims to describe the bone niche of chondrosarcoma, its role in tumour growth and drug resistance, and its clinical interest as a therapeutic target.

## 2. The Bone Niche Is Composed of Heterogeneous Cell Types with Coupled Activities

In 2003, two research laboratories demonstrated that osteoblasts formed an osteoblastic niche to sustain hemopoiesis [11, 12]. Osteoblasts establish an “epithelial-like” tissue which physically interacts with hemopoietic stem cells and contributes to their maintenance in a quiescent stage through the interaction between Tie-2 and angiopoietin-1 [13]. Nilsson et al. showed that primitive hematopoietic cells resided close to the bone surface [14]. From these observations, the concept of bone niche has strongly evolved and has been applied to cancer stem cells [15]. Indeed, the “niche” is a functional microenvironment able to promote the emergence of cancer stem cells and to provide all factors required for their development. Naturally, this concept is well recognized in the context of hematologic malignancies such as multiple myeloma [16] or leukemia [17], and these diseases appear as a stem-cell disease with a hierarchy analogous to normal hematopoietic development. However, the bone niche is not limited to osteoblasts and during skeletal remodelling, numerous cell types (preosteoclasts, preosteoblasts, endothelial cells, macrophages, etc.) are closely located in the bone matrix and their functional coordination is a prerequisite to maintain the bone and the bone niche microarchitecture. Using three-dimensional visualizations, Andersen et al. clearly demonstrated the functional relevance of these cellular interactions in the bone niche [18]. In physiological conditions in which bone resorption and bone formation are coupled, the bone surface is always covered by canopy composed by flat cells expressing osteoblastic markers and associated with sinusoidal vessels [18]. Disruption of this canopy results in the dysregulation of the coupled bone-formation bone-resorption process and leads to a bone deficiency [18]. These very elegant observations revealed that the bone niche is composed of multiple cell entities. Macrophages also contribute to the bone niche as shown by Chang et al. [19]. Indeed, a discrete population of resident macrophages has been identified between bone lining cells within endosteum and periosteum. These osteal tissue macrophages are involved in bone dynamics by controlling osteoblast functions and, more specifically, are required for efficient osteoblast mineralization [19]. Into the bone niche, self-renewal and differentiation activity are clearly balanced as shown for hemopoietic stem cells [16, 17], and this balance is being controlled by the level of hypoxia, which modulates the interactions between tumour cells and the components of bone niche. The proliferation stage of stem cells is predominant with increased levels of oxygen and hypoxia resulting in opposite effects [20, 21]. 

The concept of bone niche is also currently discussed for solid tumours and strengthens the very modern theory of “seed and soils” proposed by Paget in 1887 in which tumour cells (“seeds”) would colonize receptive foci (“soils”) [22]. This data is supported by the fact that specific molecules (e.g., cadherin and osteopontin) contribute to the stabilization of cancer cells in bone niches mimicking the cell interactions which take place during hemopoiesis [23, 24]. Such interactions have been identified in the premetastatic niche of breast carcinoma, where carcinoma cells grow avidly in bone which stores a variety of cytokines and growth factors and thus provide an extremely fertile environment for growing cells [25, 26]. The seed and soil theory can be also envisaged for the primary bone tumours. In a recent study, we reported an unexpected local osteosarcoma relapse which occurred at the exact site of autologous fat grafts in a patient who did not present any predictive factor of local recurrence [27]. Moreover, we showed that tumour growth was promoted by fat injection using a human osteosarcoma model induced in athymic nude mice. We then demonstrated that the mesenchymal stem cells isolated from adipose tissue induced exactly the same effect, probably reactivating quiescent tumour cells locally deposited into the bone tissue [27]. A recent study reinforces this theory by presenting 8 cases of osteosarcoma development several years after benign bone tumour treatment by curettage associated with bone graft. To explain the development of “de novo” sarcomas in these patients, an attraction mechanism of mesenchymal stem cells by the scaffold has been hypothesized [28]. Although mechanisms by which cancer stem cells could drive the tumour growth are still unknown, modulation of the microenvironment by mesenchymal stem cells may interfere with the biological behavior of this cell subpopulation. Similarly, inflammatory process associated with surgery may be also responsible for the reactivation of dormant tumour cells [29, 30]. Thus, a disturbance of the microenvironment and the bone niche modifies the proliferation/differentiation program of the tumour cells.

## 3. The Bone Niche of Chondrosarcoma

**Figure 1:**
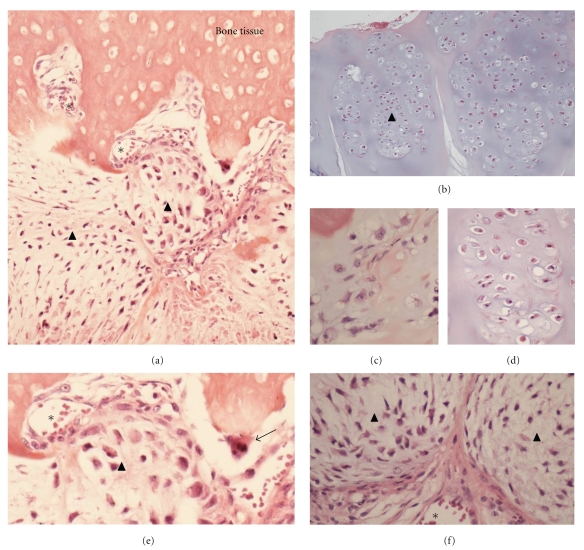
The bone niche of chondrosarcoma is composed by various cellular entities. Chondrosarcoma tissue shows heterogeneous cell morphology (a–d) with chondrocyte-like (b–d) and mesenchymal features (f). Chondrosarcoma bone niche is associated with several cell types including osteoclasts (e), endothelial cells vascularized soft tissue (f). HES staining, original magnification (×20, a and b; ×40: c–e). Tumour cells: arrow head, asterix: blood vessels, and arrow: osteoclast.

**Figure 2:**
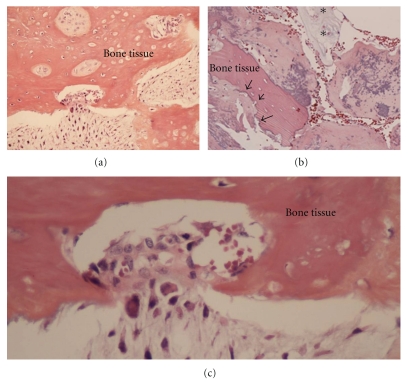
Chondrosarcoma growth is strongly linked to the bone tissue. Relationship between bone tissue and chondrosarcoma cells (a–c). Infiltration of chondrosarcoma cells into the bone tissue (permeation) (a–c). Chondrosarcoma development is associated with bone resorption foci (b). HES staining, original magnification (×20, a and b; ×40: c). Arrow: bone resorption area, arrow head: necrosis of chondrosarcoma tissue, and *: viable tumour component.

The key role of bone microenvironment in chondrosarcoma development has been suspected many years ago. Indeed, a rat intraosseous model simulating the progression of human chondrosarcoma has been set up to assess the interactions between bone environment and chondrosarcoma [31]. Transplantation of swarm rat chondrosarcoma within bone marrow or in close contact to the bone with induced periosteal lesions led to extensive bone remodelling with trabecular bone rarefaction and periosteal apposition associated with tumour growth. In contrast with these results, transplantation in close contact to the bone but without any periosteal lesion had no effect on bone, suggesting that bone healing factors interact with tumour development. The tumours which developed in intramedullary environment presented different foci with various gradings confirming that bone environment is an important factor in the pathogenesis of chondrosarcoma [31]. Histological examination of conventional chondrosarcoma reveals the presence of numerous cells types in close contact to the cartilaginous tumour cells (Figures 1(a) and 2). The morphology of cartilaginous tumour cells depends on the grading of the tumour and associated cartilage-like tissue composed by tumour chondrocytes with heterogeneous shapes (Figures 1(b)–1(e)) and tumour cell types with mesenchymal aspect (Figure 1(e)). The tumour mass is characterized by lobular foci separated by vascularized soft tissue, which establishes a continuum with bone marrow or with the surrounding tissues (Figure 1). When chondrosarcoma develops in the medullary space (central or primary chondrosarcoma), the tumour cells induce the dysregulation of the balance between osteoblasts and osteoclasts, degrading the trabecular bone, perturbing the bone marrow environment. When chondrosarcoma develops from the bone surface (peripheral or secondary chondrosarcoma), tumour mass exhibits a similar lobular morphology associated with a periosteal reaction [31]. These peripheral chondrosarcoma develop on preexisting osteochondroma defined as the most common benign bone tumours and characterized by a cartilage-capped exophytic lesion that arises from the bone cortex. Nevertheless, the limit between osteochondroma and chondrosarcoma is still unclear, especially with low-grade chondrosarcoma that is closely related to osteochondroma. These tumours interact with periosteum mimicking the “bone niche”. Periosteum is a continuous membrane intimately linked covering the bone, well vascularized and containing osteoprogenitor cells including mesenchymal stem cells [32–34]. Thereby, peripheral and central chondrosarcoma can interact with the same kind of bone microenvironment. The permeation of tumour cells into the bone tissue is associated with the activation of bone resorption through the induction of osteoclast formation (Figures 1(f) and 2). In fact, the bone niche of chondrosarcoma includes all cell types described in the other neoplastic bone diseases. The narrow relationship between chondrosarcoma cells, soft tissue, vessels, and bone cells strengthens the relevance of a specific bone niche able to sustain tumour growth. 

Can we suspect the existence of cancer stem cells in this bone niche which could be at the origin of chondrosarcoma and become quiescent in specific circumstances? Expression of SOX9 in human chondrosarcomas suggests that chondrosarcomas originate from a multipotent stem cell committed to differentiation along the chondrogenic pathway [35]. Moreover, the results of the cDNA array analyses emphasize the heterogeneous nature of chondrosarcoma. Using similar approaches, Boeuf et al. [36] proposed a new classification of chondrosarcoma in two clusters: a prechondrogenic phenotype with immature cells and a chondrogenic phenotype composed of more mature cells. Primary conventional central chondrosarcoma cells could be then grouped into two main clusters with distinctive marker expression signatures: one group clustering together with mesenchymal stem cells (CD49b-high/CD10-low/CD221-high) and a second group clustering close to fibroblasts (CD49b-low/CD10-high/CD221-low) [37]. These data strongly suggest the existence of cancer stem cells possibly with mesenchymal stem cells or fibroblast markers. Although most of the literature on chondrosarcoma has confirmed that adequate surgery is the mainstay of treatment for local tumour control, which itself constitutes a risk factor for survival, an additional feature of chondrosarcoma is also the high level of local recidive even in case of adequate surgery [38–40]. This feature is also in favour of the existence of cancer stem cells in the bone marrow which may remain dormant until some yet unknown signals promote their growth or/and metastasis formation in bone tissue. 

Hypoxia is a signal resulting in a large number of adaptive changes aimed at surviving in the hypoxic environment as well as correcting the oxygen deficit. Hypoxia inducing factor (HIF)-1 is a dimeric transcription factor composed of HIF-1 alpha and beta subunits. HIF-1 protein levels increase as a result of decreased degradation of the oxygen sensitive subunit HIF-1*α*. HIF-1 modulates changes in gene expression during hypoxia. Although the angiogenesis compound of cartilage tumours is heterogenous [41], hypoxia modulates the proliferation of chondrosarcoma cells similarly to the other solid tumour types and hemopoietic neoplasia. Thus, there is a significant relationship between the expression of HIF-1*α*, the microvessel density and the proliferating cell nuclear antigen [42]. Several authors demonstrated that malignant chondrocytes increased HIF-1*α* expression in an oxygen concentration-dependent manner and increased V-EGF expression in response to hypoxia [43–46] which is closely related to the potential malignancy of chondrosarcoma [47, 48]. Hypoxia is also known to increase chemokine receptor expression such as CXCR4 in numerous cell types [49] and CXCR4/SDF1 also indirectly promotes the proliferation and migration of tumour cells and enhances tumour-associated angiogenesis [50]. CXCR4 expressed by tumour cells contributes to their migration into the premetastatic niche [51]. Interestingly, chondrosarcoma cell invasion is increased by hypoxia-induced expression of CXCR4 and MMP1, a process mediated by HIF1*α* and ERK [52], and CXCL12, also called SDF-1, increases the invasiveness of chondrosarcoma cells [53]. Other chemokine/chemokine receptors couples are also involved in chondrosarcoma progression. Thus, the interaction of CCL5 (RANTES), a product of activated T cells present in bone environment during the tumour process with CCR5 expressed on the cell membrane enhances the migration of chondrosarcoma cells through the increase of MMP-3 production [54]. Overall, these data point out the similarities between the behaviour of chondrosarcoma cells and the invasion of leukaemia cells in the bone niche [51]. Osteopontin is also a typical example of these similarities. Indeed, osteopontin could mediate the anchoring of cancer cells in osteoblastic niches in a manner that mimics the mechanisms used by osteoblast to retain hematopoietic stem cells in these niches and to negatively regulate stem-cell pool size [55]. Osteopontin also influence the behaviour of carcinoma cells (proliferation, invasiveness, etc.) [56]. Similarly, osteopontin located in the bone matrix increases the migration and MMP expression in human chondrosarcoma and contributes to the pathogenesis of chondrosarcoma in its bone niche [57]. More recently, Vincourt et al. [58] demonstrated not only that the respective levels of C-propeptides of procollagens I and II in chondrogenic tumours but also that the interactions of chondrosarcoma cells with the surrounding extracellular matrix may modulate tumour progression, angiogenesis, and metastasis. C-propeptides of procollagen I favor angiogenesis and tumour progression, whereas C-propeptides of procollagen II exert antitumour and antangiogenic properties through apoptosis induction when they are immobilized, and progression and metastasis when they are soluble [58]. Endostatin derived from collagen XVIII, a potent endogenous antiangiogenic factor that induces regression of various tumours of epithelial origin, prevents the chondrosarcoma growth via its potential activity on endothelial cells [59]. These results demonstrate that bone microenvironment and extracellular matrix establish a very complex bone niche adapted to the tumour progression. 

The interactions between the extracellular matrix of bone niche and chondrosarcoma cells are tightly controlled by cytokines and growth factors produced by the environmental cells (osteoblasts, endothelial cells, macrophages, lymphocytes, etc.) and also by tumour cells themselves [60]. Proinflammatory cytokines are particularly associated with the pathogenesis of chondrosarcoma. Interleukin (IL)-1 regulates the expression of a disintegrin and metalloproteinase with thrombospondin motifs 1 (ADAMTS1) and VEGF by chondrosarcoma cells, then contributing to a strong positive impact of IL-1 on vascularization and tumour progression [61]. TNF-*α*, another proinflammatory cytokine, induced MMP-12 expression in chondrosarcoma cells when chondrocytes undergo malignant transformation [62] and increased also MMP-13 [63]. Members of TGF-*β* superfamily play also a crucial role in migration and metastasis of human chondrosarcoma. For instance, TGF-*β*1 and BMP-2 increase motility of human chondrosarcoma via the PI3K/Akt pathway [64, 65]. Oncostatin (OSM), a member of IL-6 cytokine family, induces a hypertrophic differentiation, with reduced SOX9 and induced Cbfa1, Coll10, MMP13, VEGF, and RANKL expression in chondrosarcoma cells. RANKL being a pro-osteoclastogenesis factor and then a proresorptive factor, OSM enhances osteoclast formation at the tumour/bone interface and reduces the ectopic bone neoformation [66].

## 4. The Bone Niche: A Sanctuary for the Drug Resistance and a Source of New Therapeutic Targets

Although bone niche represents an adequate microenvironment for the survival/proliferation of cancer stem cells and has been identified as a major parameter regulating the metastatic process [67], recent studies also described the tumour microenvironment as a sanctuary contributing to the phenomenon of drug resistance [68]. The process of drug resistance has been shown to be mediated through (i) soluble factors such as cytokines or adhesion molecules constituting *de novo* drug resistances or (ii) acquired drug resistance linked to resistance mechanisms caused by selective pressure of chemotherapy or other therapeutic drugs [68]. Chondrosarcomas are poorly vascularized in correlation with resistance to systemic chemotherapy and exhibit poor metastatic potential. However, although this poor vascularization represents a first explanation for the drug resistance, the bone niche also contributes to this resistance as observed for other tumour entities. In this context, a better definition of bone niche leads to the identification of relevant drug targets to improve the efficiency of the current treatment. This concept has been already validated in leukemia [69]. In sarcomas, similar approaches have been also envisaged [70]. Targeting of angiogenesis has been assessed in combination of chemotherapy and induced tumour necrosis [71]. Cyclooxygnease-2 (COX-2), a mediator of angiogenesis, is expressed in malignant cartilaginous tumours [72]. In chondrosarcoma, the use of celecoxib, a COX-2 inhibitor, first results in a decrease in tumour volume followed unfortunately by a relapsed tumour growth after 6 weeks [73]. Higher doses of COX-2 may be used, or a combinatory therapy based on this concept may be designed. HDAC4 represses VEGF expression and associated angiogenesis in chondrosarcoma [74]. Similarly, a therapeutic approach of chondrosarcoma based on HDAC inhibitor administration may be interesting [75, 76]. Bisphosphonates and rapamycin and its derivatives have been originally developed, respectively, as antiresorptive and antifungal agents [77, 78]. However*, in vitro* and *in vivo* experiments demonstrated that these compounds are multifunctional molecules exerting their effects not only on bone remodelling but also on tumour cell growth. mTOR targeting has been envisaged for numerous cancer types including malignant primary bone tumours [78–80], and a very impressive response of myxoid chondrosarcoma has been obtained in combination with cyclophosphamide [81]. The main targets of bisphosphonates are bone-resorbing osteoclasts [82] which contribute to the hemopoietic and tumour bone niche [82]. Bisphosphonates also reduce the proliferation and invasion of chondrosarcoma [83, 84]. In preclinical model of chondrosarcoma, zoledronic acid slows down rat primary development and recurrent tumour progression after intralesional curettage and increases overall survival [85]. Thus, osteoclasts targeting may be used in prevention of chondrosarcoma recurrence. Cytokinic treatment represents another relevant therapeutic approach of chondrosaroma [2]. Oncostatin M, a member of the IL-6 cytokine family mainly produced by macrophages, neutrophils, and T lymphocytes, is a cytostatic factor for chondrosarcomas *in vitro* and *in vivo *[66]. This growth inhibitory effect is also observed with two other cytokines of the same family able to reduce chondrosarcoma expansion but with a lower efficiency: IL-6 in association with its soluble receptor and IL-27 [66]. This list is not exhaustive but gives some evidence of the interest to target or to modulate the bone niche components to improve chondrosarcoma treatment.

## 5. Conclusion

The treatment of chondrosarcoma is currently based on surgery, radiotherapy, and chemotherapy being occasionally used for metastatic tumours. However, a recent concept has emerged based on the key role played by the tumour microenvironment in the tumour invasiveness and in the drug-resistance phenomenon. This bone niche allows to identify new therapeutic targets for chondrosarcoma, and it appears clearly that a better understanding of the chondrosarcoma bone niche will open nonsurgical therapeutic options for chondrosarcoma which could also be combined with surgery.
